# Evaluation of Factor VIII as a Risk Factor in Indian Patients with DVT

**DOI:** 10.1155/2015/307879

**Published:** 2015-09-01

**Authors:** Darpanarayan Hazra, Indrani Sen, Edwin Stephen, Sunil Agarwal, Sukesh Chandran Nair, Joy Mammen

**Affiliations:** ^1^Department of Vascular Surgery, The Christian Medical College, Vellore 632004, India; ^2^Department of Transfusion Medicine and Immunohaematology, The Christian Medical College, Vellore 632004, India

## Abstract

*Introduction*. Elevated factor VIII population in the Indian population has not been studied as a possible risk factor for deep vein thrombosis (DVT). High factor VIII level is considered a predisposing factor for DVT and its recurrence. However it is known to vary between populations and its exact role in the etiopathogenesis of thrombophilia remains unknown. *Material and Methods*. Factor VIII levels of patients with DVT who had undergone a prothrombotic workup as a part of their workup was compared to normal age matched controls in a 1 : 3 ratio. *Results*. There were 75 patients with DVT who had undergone a prothrombotic workup in the course of their treatment for lower limb DVT. In these, 64% had levels of factor VIII more than 150 as compared to 63% of normal controls (*p* > 0.05, not significant). *Conclusion*. Elevated factor VIII in the Indians may not be associated with the same thrombotic risk as seen in the West. We find a variation in the levels of factor VIII with a different “normal” than what is reported in other populations. This needs further study to elucidate the role of factor VIII in the evaluation and treatment of thrombophilia.

## 1. Introduction

High factor VIII level is considered a predisposing factor for deep vein thrombosis (DVT) and its recurrence. Koster et al. in the Leiden Thrombophilia study reported that elevated levels above 150 IU/dL carried a 5-fold increased risk for DVT [1]. However, factor VIII levels can vary due to a multitude of physiological and disease factors. Normal levels also vary between populations, with higher plasma levels of factor VIII reported from Africa and Japan [2–4]. Many population differences in thrombophilic risk factors are reported in India; however, factor VIII levels have not yet been studied [5–11]. This is the first study to analyze whether a high factor VIII level in Indian patients with DVT was a significant prothrombotic risk factor for venous thromboembolism.

## 2. Materials and Methods

We designed a retrospective case control study comparing factor VIII levels in a population with DVT to normal controls.

Inclusion criteria were as follows: factor VIII levels from patients who had undergone a thrombophilia workup as a part of management of deep vein thrombosis. Management is in accordance with the ACCP guidelines [12]. A thrombophilia workup is ordered when indicated in patients with an initial episode of unprovoked DVT after the initial treatment phase is over. The patients are not on anticoagulation for at least 6 weeks at the time of testing. Patients with obvious provoking factors or those who are planned for lifelong anticoagulation are not offered thrombophilia testing. Exclusion criteria were as follows: children, pregnant women, and patients with thrombosis of the visceral, cerebral, or pulmonary venous beds were excluded as levels of factor VIII are known to vary in these conditions. Controls were as follows: blood samples from healthy donors were used as controls. There was no history of thrombosis in the control group that was matched for baseline characteristics like age and sex. The design was formulated in discussion with the biostatistics department. As it was mainly a numerical analysis of data, institutional review was not required.

Thrombophilia workup included blood cell counts, coagulation parameters (PT with INR, aPTT), factor levels, d-dimer, thrombin time, fibrinogen levels, proteins C and S, antithrombin, lupus anticoagulant, DDVRT, Ham's test, sucrose lysis, sickle test, homocysteine levels, and Thromboelastogram. Genetic markers of thrombosis were studied when indicated. The “normal” cut-off value used for factor VIII was 150 IU/dL [1]. Data was analysed from a prospectively maintained hospital registry.

### 2.1. Method of Factor VIII Assessment

All samples were collected in the laboratory using an evacuated tube system, Vacuette (Greiner) into citrate anticoagulant at the ratio of 1 : 9 following the proper order of draw, and processed with 30 minutes of the collection after double centrifugation. FVIII : C is assayed with 1-2 hrs of the sample collection and for rest of the assays like AT, PC, and PS the citrated samples are frozen in vials and stored at −800°C.

The calibrators have an assigned value traceable to the international standard and the value of the IL calibrator has been verified on the CS2000i after being tested on ACL Top analyser that the laboratory has and this is further validated against 6th World Health Organisation (WHO) International Standard (IS) for factor VIII/VWF plasma (07/316) randomly as we have it in our inventory since our laboratory is enlisted as one of the 22 reference laboratories to assign values to the IS by NIBSC, UK. Any sample which is closest to the samples being tested with no matrix effect is a good reference material and can be used as a precision tool like a control or even as a calibrator (as long as it has an assigned value traceable to a standard), providing that it is stable across days it is being tested. At a temperature of −800°C coagulation factors are stable for at least a period of 6 months. The PNP prepared in our laboratory is stored at −800°C and is prepared once in 4 months as it gets exhausted. It has proven to be a good precision tool like the commercial control that we run along. Only FVIII : C activity assay is done and FVIII : Ag is not performed as part of the thrombophilia screen.

### 2.2. Statistical Methods

The sensitivity and specificity were calculated for every cut-off value including the normal. As the design is matched case control design, we have used McNemar's chi-square test to test association between factor VIII in cases and factor VIII in controls. The *p* value was fixed at 0.05, 5% level of significance. The ROC curve was drawn by taking false positive rate at the *x*-axis and the sensitivity at the *y*-axis. R software was used to draw the ROC curve. Data was entered using EPIDATA software and analyzed using SPSS and R software.

## 3. Results

There were 75 patients with DVT who had undergone a prothrombotic workup in the course of their treatment for unprovoked lower limb DVT. The average age was 40.2 years with a range of 22–64 years. The male : female ratio was 4 : 1. The results of the thrombotic workup were normal in 13% and were positive for lupus anticoagulant in 23% and elevated factor VIII in 56%, with more than one prothrombotic risk factor identified in 8%. Healthy blood donor's samples were analysed for plasma factor VIII as controls (*n* = 289). There was no history of thrombosis, the control group which was matched for baseline characteristics like age and sex.

Amongst cases, 48 patients (64%) had elevated levels of factor VIII (>150); of these, elevated factor VIII was the only abnormality in 42 (56%). In the control population (*n* = 289), 181 patients (63%) had factor VIII levels above the normal cut-off value.

At the cut-off of 150, a 2 × 2 table was generated to determine the association between factor VIII and the presence of DVT (Table 1). This provided a sensitivity of 64% and specificity of 63%, *p* < 0.05 (not significant). As both the sensitivity and specificity at the presently used normal were poor, we calculated these statistics for other proposed (higher and lower) cut-offs (Table 2). The *p* values at all these proposed cut-offs were calculated; none reached significance.

**Figure 1:**
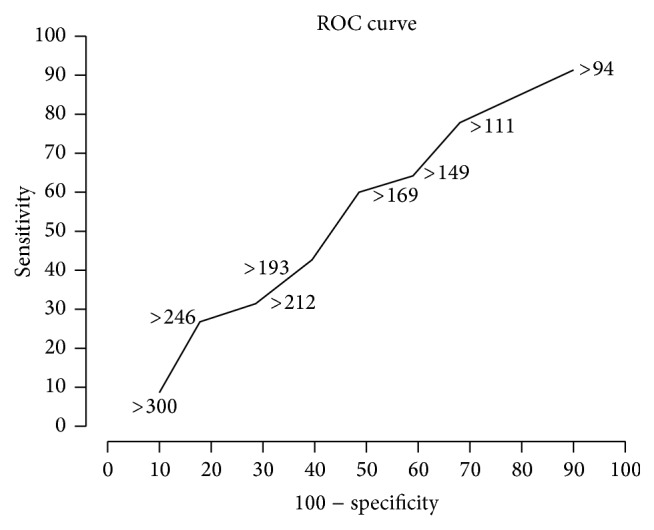
ROC curve-Factor VIII, no relevant cutoff.

ROC analysis was plotted to assess whether a higher factor VIII level is necessary as a cut-off in our population (as 63% of controls with no evidence of thrombosis also had elevated plasma levels of factor VIII). This was done to check the diagnostic accuracy of 150 IU/mL as the clinically relevant cut-off (not factor VIII levels as a diagnostic tool for DVT). However, the plot yielded a flat curve without a clinically significant cut-off test result level (Figure 1). This demonstrated that we could not determine a clinically relevant cut-off to determine a “high” factor VIII in our population.

## 4. Discussion

The relative risk for venous thrombosis of factor VIII : Ag levels ≥150 IU/dL is 5.3 (95% CI 2.7 to 10.1) compared with levels <100 IU/dL, which is very similar to the risk previously reported for factor VIII activity levels ≥150 IU/dL [1]. Population studies in the African Americans and Japanese have demonstrated that plasma FVIII levels are influenced by ethnicity, with higher levels compared to Caucasians [2–4]. In the studied populations, increased levels above 150 IU/dL have been shown to cause a dose-dependent risk increase in VTE and its recurrence, both when present as an isolated risk factor or in combination with other prothrombotic states [1]. The biological pathway in causation of thrombosis remains unknown [13–16]. In clinical practice we find that our population also has increased plasma FVIII levels but there is no published data on this topic from India. Though most centres include this as a part of screening for thrombophilia, there is considerable variation in interpretation of results with regard to recurrence and further treatment.

The results of the prothrombotic workup for venous thrombosis in our population differ from what is commonly seen in the West [17]. The prevalence of heterozygosity for other prothrombotic risk factors also varies: for example, the factor V Leiden mutation in Indian, Arab, Canadian, and Israeli populations is lower (1 to 8.5%) than European studies (5–8%). Factor II abnormalities are not as common in our patients [5–11]. Regional variations, for example, higher APC mutations from studies done in the Northern and Western parts of the country, are also reported [5–11].

The commonest abnormality seen in our study was elevated factor VIII levels followed by lupus anticoagulant positivity. This is the first study from India which reports this pattern. We believe that the difference in our study is due to population variation in the levels of factor VIII. There is a remote possibility that this difference in the underlying prothrombotic condition is related to the site of occurrence of DVT; this needs further study [1]. Our study found a relatively “high” blood level of factor VIII in normal controls. Again, we attribute this to population variation. This is a relevant finding; such a variation has not been previously reported in India.

Factor VIII or antihaemophilic factor is perhaps better studied in haemophilia. However elevated levels are also known to cause venous and occasionally arterial thrombosis (coronary, cerebrovascular beds). It is considered a prothrombotic risk factor along with factor V Leiden, prothrombin 20210A, elevated fibrinogen, antithrombin/protein C/protein S deficiency, hyperhomocysteinemia, and lupus anticoagulant positivity [1, 18, 19]. Factor VIII circulates bound to von Willebrand factor (vWF). The blood levels are influenced by various patient and disease factors. Patient factors include age (increase of 5 IU/dL with each decade increase in age), sex (higher in females), pregnancy, race, blood group, and genetic factors affecting vWF levels. However, no direct genetic factors affecting factor VIII levels are reported. Obesity, diabetes, insulin, liver disease, increased triglycerides, endothelial stimulation (exercise, V2 receptor analogs, and surgery), fibrinogen, intravascular haemolysis, chronic inflammation, malignancy, hyperthyroidism, and renal disease also cause increase in factor VIII levels [13, 20]. It is considered a risk factor for DVT in women using oral contraceptives; OCPs themselves do not influence factor VIII levels. The estimated population-attributable risk for factor VIII levels ≥150 IU/dL is ≈16% [1, 13]. This makes it an important prothrombotic risk factor. Thus, treatment parallels the other thrombophilias in which a specific genetic mutation is identified [1, 12]. There are no treatment guidelines specific to elevated factor VIII levels especially where the normal population demonstrated increased baseline plasma levels like in our population. Hence identification of a prothrombotic risk factor implies lifelong anticoagulation; this is a major clinical decision and influences all aspects of the patient's life [18, 19]. However, treatment based on assumption of similar “normal” blood levels among populations may be erroneous. The clinical behaviour in the patients with “elevated” levels therefore may not reflect a true prothrombotic tendency and may overtreat this patient group and cause undue anxiety. A cut-off level of 150 IU/dL may not be significant to diagnose elevated factor VIII levels as a prothrombotic risk factor in our population. There is a need for formulation of population specific treatment protocols.

## 5. Limitations

This study fails to find a suitable cut-off for a “normal” plasma factor VIII level: this is probably due to a small sample size with data from a single centre. The retrospective design and testing factor VIII at a single point are other limitations. The design comparing the levels of FVIII between the patients with unprovoked DVT and healthy people, though not easily justified, is used to highlight the lack of published data in Indian patients. We do not have prospective data to ascertain whether any of the healthy controls with elevated FVIII levels develop an unprovoked DVT during their life. This is also a serious limitation of our study.

## 6. Conclusion

Elevated factor VIII in the Indians may not be associated with the same thrombotic risk as seen in the West. We find a variation in the levels of factor VIII with a different “normal” than what is reported in other populations. This needs further study to elucidate the role of factor VIII in the evaluation and treatment of thrombophilia.

## Figures and Tables

**Table 1:** Factor VIII distribution in cases and controls using 150 (IU/dL) as cut-off.

	Factor VIII levels (IU/dL)	
	>150	<150
DVT cases, *n* = 75	48 (64%)	27 (36%)
Controls, *n* = 289	181 (63%)	108 (37%)

**Table 2:** Sensitivity and specificity value of VIII at different cut-off levels.

Factor VIII cut-off value	Cases (*n*)	Control (*n*)	Sensitivity (%)	Specificity (%)	*p* value
>149	49	169	64.5	40.9	Ns
<149	27	117			

>169	41	139	59.9	51.4	Ns
<169	35	147			

>193	32	112	42.1	60.8	Ns
<193	44	174			

>212	24	82	31.6	71.3	Ns
<212	52	204			

>246	20	51	26.3	82.2	Ns
<246	56	235			

≥300	7	29	9.2	89.9	Ns
<300	69	257			
